# Lactation duration and lifetime progression to metabolic syndrome in women according to their history of gestational diabetes: a prospective longitudinal community-based cohort study

**DOI:** 10.1186/s12967-023-04005-w

**Published:** 2023-03-06

**Authors:** Maryam Farahmand, Maryam Rahmati, Fereidoun Azizi, Fahimeh Ramezani Tehrani

**Affiliations:** 1 Reproductive Endocrinology Research Center, Research Institute for Endocrine Sciences, Shahid Beheshti University of Medical Sciences, Tehran, Iran; 2 Endocrine Research Center, Research Institute for Endocrine Sciences, Shahid Beheshti University of Medical Sciences, Tehran, Iran

**Keywords:** Metabolic syndrome, Gestational diabetes mellitus, Lactation, Hypertension, Insulin resistance

## Abstract

**Background:**

Despite the many signs of progress in pharmacotherapies, metabolic syndrome (MetS) is one of the main public-health burdens worldwide. Our study aimed to compare the effect of breastfeeding (BF) in women with and without gestational diabetes mellitus (GDM) on MetS incidence.

**Methods:**

Of females who participated in the Tehran Lipid and glucose study, women who met our inclusion criteria were selected. The Cox proportional hazards regression model, with adjustment of potential confounders, was done to evaluate the relationship between duration of BF and incident of MetS in women with a GDM history compared to non-GDM.

**Results:**

Out of 1176 women, there were 1001 non-GDM and 175 GDM. The median follow-up was 16.3 (11.9, 19.3) years. Results of the adjusted model illustrated that the total BF duration was negatively associated with MetS incidence risk (hazard ratio (HR) 0.98, 95% CI 0.98–0.99) in total participants indicating that per one-month increase of BF duration, the hazard of MetS reduced by 2%. The HR of MetS in Comparison between GDM and non-GDM women demonstrated significantly more reduced MetS incidence with a longer duration of exclusive BF (HR 0.93, 95% CI 0.88–0.98).

**Conclusions:**

Our findings illustrated the protective effect of BF, especially exclusive BF, on MetS incidence risk. BF is more effective in reducing the risk of MetS among women with a history of GDM than among women without such a history.

**Supplementary Information:**

The online version contains supplementary material available at 10.1186/s12967-023-04005-w.

## Introduction

Metabolic syndrome (MetS) is a group of risk factors that enhance the chance of increasing some diseases, especially heart disease. MetS is now considered a "developmental origin of health and disease", which, on the one hand, can have its origins in early life; on the other hand, early interventions can have the potential impact on preventing MetS [1]. Precise identification of influential factors in preventing and controlling the increasing incidence and prevalence of MetS is crucial [1]. As mentioned, using professional factors or interventional programming to prevent or treat MetS is needed and beneficial.

The World Health Organization (WHO) feeding recommendation is that infants at six months of age receive exclusive breastfeeding (BF) without receiving complementary foods, and then BF should be accompanied by complementary foods [2]. BF duration per se is a critical period in women's reproductive process, and it has many benefits for both mother and child. Thereby, BF can improve health across two generations. There is a piece of solid evidence on the advantages of BF for children later in life. However, the effects of BF on the mother's health have usually been overlooked [3]. BF may result in the improvement of the physical and emotional health situations of the mother during her lifetime. The beneficial mental and physical effects of BF are divided into two groups: early and late effects [4]. Reduced adiposity, weight, stress, and anxiety are examples of early effects [5–7]. Furthermore, reducing some noncommunicable diseases (NCDs) such as cardiovascular diseases (CVDs), rheumatoid arthritis, and Alzheimer's are instances of late effects of BF on women's health [8–10].

Today, what should be more considered is finding prolonged beneficial effects of BF for mothers. Still, relatively few studies have been done to assess the effect of BF on women's health during their lifetimes [11]. A recent meta-analysis (2020) reported inconclusive results regarding the association between the duration of BF and MetS [12]. Moreover, the association between BF and MetS may be influenced by mothers' histories of gestational diabetes mellitus (GDM). GDM is stated as "diabetes diagnosed in the second or third trimester of pregnancy that is not clearly overt diabetes" [13] and occurs in nearly 13% of births in 2019 [14].

On the one hand, studies illustrated a higher thyroid hormone level in women with a history of GDM with a longer BF duration may have a potential beneficiary effect on their cardiometabolic status through a reduction in weight gain [15]. On the other hand, lactation due to various maternal and fetal morbidities secondary to GDM may cause postponed lactogenesis and the infants' poor sucking type [16, 17]. So further research should be performed to evaluate the beneficial roles of BF duration, for instance, as a preventive factor, on diseases such as MetS [12].

To address these gaps, we aimed to explore the effects of BF and its duration on women according to their history of GDM in a group of women using a longitudinal prospective community-based method with nearly two decades of follow-up.

## Methods

### Research design and subjects

**Fig. 1:**
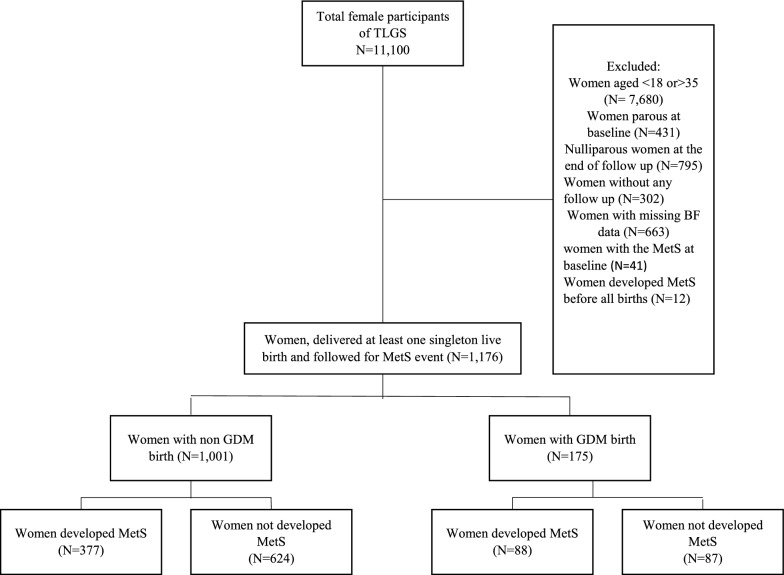
Study flowchart. TLGS, Tehran lipid & glucose study; GDM, gestational diabetes Mellitus; MetS, Metabolic syndrome; BF, breastfeeding

The present study subjects were selected using the baseline information of the 11,100 Tehran lipid and glucose study (TLGS) female participants. The TLGS is an ongoing prospective study initiated in 1998 to determine the prevalence and incidence of NCDs risk factors. Details of TLGS have been published elsewhere [18]. To perform the current study, prospectively, women were followed who were nulliparous and without metabolic disease at recruitment. Of the 11,100 female participants in the TLGS study, there were 7680 of childbearing age (18–35 years). Of these were excluded women who did not give birth during the study period (N = 784). As well, women that were diagnosed with MetS before their first pregnancy (N = 41), parous women at baseline  (N = 431), those that had a multiple birth pregnancy (N = 11), and those without BF (N = 663) or follow-up data (N = 302) were excluded. Furthermore, since we aimed to report the incidence of MetS during women's lifetimes after BF duration, we excluded those who experienced MetS before their first BF duration (N = 12) (Fig. 1), leaving a sample of N = 1176 for analysis.

### Measurements

All participants' data were gathered and measured at exams almost every three years. Face-to-face interviews were performed to collect socio-demographic variables and data on several risk factors for NCDs and reproductive histories by trained staff.

For all participants, standardized procedures and calibrated equipment were used to measure weight and height with minimal clothing, without shoes in a standing position. Calculating the body mass index (BMI) was done by dividing weight in kilograms by height in meters squared (kg/m^2^). Hip circumference was measured via an unstretched measuring tape to the nearest 0.1 cm. Waist circumference (WC) was measured centrally between the lower rib margin and the iliac crest at the side of the umbilicus at the end of a gentle expiration. Blood pressure (BP) was measured twice in the right arm after a 15-min rest in a sitting position and was evaluated according to the mean of the two measurements. Physical activity was assessed by the modifiable activity questionnaire. The participants were questioned about the physical activities they had engaged in over the previous 12 months. So that participants with fewer than 600 metabolic equivalent task minutes per week were categorized as a low physical activity group [19, 20]. Blood samples were gathered after a 12- to 14-h overnight fasting (between 7:00 and 9:00 a.m.). Other details for laboratory measurements were published elsewhere [18].

### Definitions

#### Exposures

The duration of BF has been reported by the response of the samples in months. The total cumulative BF duration for each subject was calculated by collecting all BF months. The duration of BF (as an exposure variable) was calculated in multiparous women who had previously BF and developed MetS in their next pregnancy based on the total duration of BF before the event of MetS. Participants reported exclusive BF duration and the duration of continuing BF accompanied by complementary foods, named partial BF, by answering two questions separately. According to the world health organization (WHO) definition, at first, after delivery, infants, until (maximum) six months of age, receive exclusive BF without receiving complementary foods. Also in Iran, the definition of exclusive BF is only giving breast milk to the infant till the end of 6 months after birth, minus giving any solid food or other liquid, even water, except for vitamin drops or syrups, mineral supplements, and medications prescribed by the doctor [21, 22]. Then partial BF contains BF should be accompanied by complementary foods gradually [23].

GDM was defined as any degree of glucose intolerance with beginning or first recognition during pregnancy [24]. Participants reported a history of GDM. The sensitivity and specificity of self-reported GDM have been revealed to be 100% and 92%, respectively [11].

#### Outcome

MetS was defined according to the Joint Interim Statement [25], as the presence of any three out of the five following risk factors: (1) Abdominal obesity: WC ≥ 90 cm, accordingly population- and country-specific cutoffs for Iranians [26], (2) fasting plasma glucose (FPG) ≥ 100 mg/dL or drug treatment, (3) Fasting triglycerides (TG) ≥ 150 mg/dL or drug treatment; (4) Fasting high-density lipoprotein cholesterol (HDL-C) < 50 mg/dL in women or drug treatment and (5) Elevated blood pressure (BP) defined as systolic blood pressure (SBP) ≥ 130 mm Hg, diastolic blood pressure (DBP) ≥ 85 mmHg or antihypertensive drug treatment.

### Statistical methods

Continuous variables were checked for normality based on the one-sample Kolmogorov–Smirnov test. They were presented as mean (standard deviation) if they had a normal distribution or median with an interquartile range (IQR25–75) for variables with skewed distribution. Categorical variables were presented as numbers and percentages. Demographic and clinical characteristics of women were compared according to GDM/or MetS status using the student t-test or chi-square test for continuous or categorical data, respectively. The Mann–Whitney test was applied to compare variables with skewed distribution.

Data collection for this investigation began in January 1999 and ran until March 2022, with follow-up periods of three years. There has been data collection for a cumulative median follow-up of 16.3 years (interquartile range: 11.9–19.3). We applied the Cox regression model to assess the hazard ratios (HRs) and 95% confidence intervals (CIs) for BF (exclusive, partial, and total) association with MetS in participants. Initially, all risk factors were included in the univariate model. Next, variables, which were found to be significant in the univariate model (P < 0.2), were also included in the multivariate model. The survival time was considered the interval between the age at the entrance and the age (in years) when MetS was detected for the first time or in the last follow-up. Women, who did not develop MetS by the end of the follow-ups, were considered lost to follow-up or censored.

Both unadjusted and adjusted Cox regression models were applied. Confounding factors, including age, BMI, family history of diabetes mellitus (DM) at baseline, physical activity, education, and parity were entered into the multivariate Cox model (physical activity, education, and parity were considered time-dependent covariates in the model).

Interaction analysis was applied to explore if the effect of BF (exclusive, partial, and total) on the hazard of the MetS is affected by GDM status. For this purpose, an interaction term of these two (BF and GDM) was entered in the Cox regression model, and HRs as well as their 95% confidence intervals, were estimated.

As a sensitivity analysis, to eliminate the effect of menopause status during follow-up, menopausal women were excluded, and the association of BF with MetS was reassessed.

Statistical analysis was performed using the software package STATA (version 13; STATA Inc., College Station, TX, USA); the significance level was set at *p* < 0.05.

## Results

Of 1176 eligible participants, 175 (14.9%) women had a history of GDM, and 1001 (85.1%) women without a history of GDM were recruited. The median and interquartile range of follow-up time for the current analysis were 16.3 (11.9, 19.3) years. During the follow-up, 88 (50.3%) women from the GDM group and 377 (37.3%) women from the non-GDM group developed MetS. The characteristics of women according to GDM and MetS status were presented in Tables 1 and 2, respectively. Based on the potential variables in Tables 1 and 2, we considered age, BMI, family history of DM, physical activity, education, parity, and smoking in the univariate model. Among them, only the variable "smoking" with a p-value greater than 0.2 did not enter the multivariate Cox model. Among the components of MetS, women with a history of GDM had higher values of WC, FPG, and TG than non-GDM women (Table 1). All components of the MetS were significantly higher in women who were incident the MetS during the study than in non-suffering women. Lower physical activity was reported by women who were incident MetS than others (33.5% vs. 40.0%) (Table 2).

**Table 1:** Baseline characteristics of participants according to GDM history

Baseline characteristics	GDM (n = 175)	Non-GDM (n = 1,001)	p-value
Education (diploma and higher), n (%)	106 (60.6)	622 (62.1)	0.7
Family history of diabetes, n (%)	48 (27.4)	201 (20.1)	**0.03**
DM, n (%)	13 (7.6)	10 (1.0)	** < 0.001**
Age (year), mean (SD)	25.6 (4.8)	26.0 (4.9)	0.3
BMI (kg/m^2^), mean (SD)	25.3 (4.5)	24.7 (4.8)	0.09
WC (cm), mean (SD)	81.3 (10.7)	79.2 (11.6)	**0.03**
Physical activity (medium to high), n (%)	68 (38.9)	356 (35.6)	0.4
Smoking status (ever), n (%)	2 (1.1)	19 (1.9)	0.5
*Fasting*			
FPG (mg/dl), mean (SD)	92.2 (30.8)	84.9 (10.1)	** < 0.001**
HDL cholesterol (mg/dl), mean (SD)	44.5 (11.0)	45.8 (10.6)	0.1
TG (mg/dl), median (Q_25_-Q_75_)	98 (72–137)	89.5 (67–121)	**0.02**
SBP (mmHg), mean (sd)	107.1 (11.4)	105.9 (10.4)	0.2
DBP (mmHg), mean (sd)	71.1 (8.9)	71.0 (8.4)	0.9
*BF duration (months)*			
Total BF (month), median (Q_25_-Q_75_)	29 (7–48)	24 (0–42)	**0.003**
Partial BF (month), median (Q_25_-Q_75_)	22 (4–38)	18 (0–33)	**0.002**
Exclusive BF (month), median (Q_25_-Q_75_)	6 (1–10)	5 (0–10)	**0.03**
Duration of follow-up, median (Q_25_-Q_75_)	14.7 (9.6–18.1)	16.5 (12.4–19.4)	0.002

GDM, gestational diabetes mellitus; BMI, body mass index; WC, waist circumference; FPG, fasting plasma glucose; DM, diabetes mellitus; HDL, high-density lipoprotein; TG, triglyceride; SBP, systolic blood pressure; DBP, diastolic blood pressure; BF, breastfeeding

p-value < 0.05 is statistically significant

**Table 2:** Baseline characteristics of participants according to MetS status

Baseline characteristics	Incident MetS case participants (n = 465)	Non-case participant (n = 711)	p-value
Education (diploma and higher), n (%)	273 (58.7)	455 (64.0)	0.07
Family history of diabetes, n (%)	115 (24.7)	134 (18.8)	**0.02**
Physical activity (medium to high), n (%)	186 (40.0)	238 (33.5)	**0.02**
Smoking status (ever), n (%)	10 (2.1)	11 (1.5)	0.4
DM, n (%)	21 (4.6)	2 (0.3)	** < 0.001**
GDM status (ever), n (%)	88 (18.9)	87 (12.2)	**0.002**
Age (year), mean (SD)	27.2 (4.9)	25.1 (4.7)	** < 0.001**
BMI (kg/m^2^), mean (SD)	27.1 (5.2)	23.2 (3.8)	** < 0.001**
WC (cm), mean (SD)	85.3 (12.1)	75.7 (9.3)	** < 0.001**
SBP (mmHg), mean (SD)	108.4 (10.9)	104.5 (10.0)	** < 0.001**
DBP (mmHg), mean (SD)	73.1 (8.7)	69.6 (8.0)	** < 0.001**
*Fasting*			
FPG (mg/dl), mean (sd)	89.0 (21.6)	84.1 (8.5)	** < 0.001**
HDL cholesterol (mg/dl), mean (SD)	43.4 (10.8)	47.1 (10.3)	** < 0.001**
TG (mg/dl), median (Q_25_-Q_75_)	116 (85–151)	79 (62–101)	** < 0.001**
*BF Duration (months)*			
Total BF, median (Q_25_-Q_75_)	24 (0–42)	24 (0–44)	0.4
Partial BF, median (Q_25_-Q_75_)	18 (0–31)	19 (0–35)	0.2
Exclusive BF, median (Q_25_-Q_75_)	5 (0–10)	5 (0–10)	0.8

MetS, metabolic syndrome; BMI, body mass index; WC, waist circumference; FPG, fasting plasma glucose; DM, diabetes mellitus; HDL, high-density lipoprotein; TG, triglyceride; SBP, systolic blood pressure; DBP, diastolic blood pressure; BF, breastfeeding

p-value < 0.05 is statistically significant

Table 3 summarizes the results of the Cox regression analysis regarding the association between the BF and the hazard of MetS. There are three models reported in Table 3. To eliminate the influence of collinearity, total, partial, or exclusive BF is independently incorporated into each model. Adjusted Cox regression analysis showed that an increase in total BF per month was related to a 2% decrease in the risk of MetS incidence (HRadj: 0.98, 95% CI: 0.98–0.99, p-value = 0.001). Moreover, a per-month increase in exclusive BF was associated with a 3% lower risk of MetS incidence (HRadj: 0.97, 95% CI: 0.95–0.98, p-value = 0.03).

**Table 3:** Unadjusted and multivariable-adjusted* Cox regression analysis for the effect of BF on hazards (95% CIs) of incident MetS

BF duration	Unadjusted model		Adjusted model	
	HR (95% CI)	p-value	HR (95% CI)	p-value
Total BF	0.99 (0.98–0.99)	**0.01**	0.98 (0.98–0.99)	**0.001**
Partial BF	0.98 (0.97–0.99)	**0.01**	0.99 (0.98–0.99)	** < 0.001**
Exclusive BF	0.97 (0.96–0.99)	**0.02**	0.97 (0.95–0.98)	**0.031**

Adjusting variables were age, BMI, family history of diabetes at baseline and physical activity, education, and parity

Physical activity, education, and parity were included in the model as time-dependent covariates

MetS, metabolic syndrome; BF, breastfeeding

p-value < 0.05 is statistically significant

Table 4 reports the result of the interaction analysis to explore the interaction effect of GDM and BF duration (exclusive, partial, and total) on the incidence of MetS. The only significant interaction effect was revealed with exclusive BF in both unadjusted and adjusted models. The adjusted model showed that an increase in exclusive BF per month in women with a history of GDM decreased the hazard of MetS incidence by 7% compared to non-GDM women (HRadj: 0.93, 95% CI: 0.88–0.98, p-value = 0.01).

**Table 4:** Interaction analysis for the association between BF*GDM and hazard ratio of MetS

Variable	Unadjusted model		Adjusted model	
	HR (95% CI)	p-value	HR (95% CI)	p-value
GDM	2.23 (1.53–3.26)	< 0.001	2.21 (1.53–3.24)	< 0.001
Total BF	0.99 (0.98–0.99)	0.03	0.99 (0.97–0.99)	0.005
Total BF* GDM	0.99 (0.97–1.01)	0.1	0.98 (0.98–1.01)	0.07
GDM	2.08 (1.44–2.99)	< 0.001	2.06 (1.43–2.97)	< 0.001
Partial BF	0.99 (0.98–0.99)	0.04	0.99 (0.98–0.99)	0.003
Partial BF * GDM	0.99 (0.97–1.01)	0.2	0.99 (0.97–1.01)	0.1
GDM	2.42 (1.68–3.50)	< 0.001	2.49 (1.72–3.61)	< 0.001
Exclusive BF	0.98 (0.96–1.01)	0.1	0.98 (0.96–1.01)	0.20
Exclusive BF* GDM	0.94 (0.89–0.99)	**0.02**	0.93 (0.88–0.98)	**0.01**

Adjusting variables were age, BMI, family history of DM at baseline and physical activity, education, and parity

Physical activity, education, and parity were included in the model as time-dependent covariates

The reference group is non-GDM

MetS, metabolic syndrome; BF, breastfeeding; GDM, gestational diabetes mellitus

p-value < 0.05 is statistically significant

The results of subgroup analysis for exploring the effect of BF on the hazard of MetS in those with and without GDM are presented as Additional file 1: Table S1. We found that the hazard ratio per month of BF has a protective effect on MetS in both groups of GDM (total or partial BF), except for exclusive BF, which was not statistically significant in the non-GDM subgroup.

During follow-up, 181 (15.4%) women went through menopause. Additional file 1: Table S2, S3 show the HR (95% CI) of MetS for the effect of BF duration in non-menopausal women. Additional file 1: Table S2 reveals BF duration types in all nonmenopausal participants statistically significantly decreased the risk of MetS incidence (p-value < 0.05). Additional file 1: Table S3 shows that the interaction between BF duration and GDM was significant for BF duration (exclusive, partial, and total).

In non-menopausal women, per month increase in total BF decreased the risk of MetS in GDM women by 2% (HRadj: 0.98, 95% CI: 0.96–0.99, p-value = 0.01); 3% reduction in the risk of MetS was observed for GDM women with increasing one month partial BF (HRadj: 0.97, 95% CI: 0.96–0.99, p-value = 0.02); and was shown in exclusive BF for GDM women was associated with decreasing 9% in hazard of MetS (HRadj: 0.91, 95% CI: 0.85–0.97, p-value = 0.004) compared to non-GDM women.

## Discussion

Our findings illustrated that the duration of BF was associated with a lower risk of MetS incidence even after adjusting for potential confounders, including age, BMI, family history of DM at baseline, physical activity, education, and parity. Each month of the BF duration reduced the incidence of MetS by 2%. Furthermore, a longer duration of exclusive BF further reduced the risk of MetS incidence in women, especially in those with a history of GDM (by 7% each month of exclusive BF) after adjusting for potential confounders.

Several studies have reported the beneficial effects of lactation on maternal cardiometabolic blood profiles [12]. A few studies investigate the effect of BF duration on metabolic syndrome compared to women with a history of GDM and non-GDM via a longitudinal study [11]. In agreement with our study, Gunderson et al. reported a stronger protective association for each month of BF with the incidence of the MetS, which was 39–56% for non-GDM women and 44–86% for GDM women [11].

Several mechanisms may partly explain the beneficial effect of BF on the MetS and its components.

The prolactin increase begins during pregnancy, reaches peak concentration level till term, and remains above nonpregnant women's level with pulsatile secretion up to weaning [27]. Previous experimental research on prolactin receptor knockout mice revealed a physiological role in pancreatic islet formation and function for prolactin [28]. The pathogeneses of MetS pertaining to pregnancy and BF may be partially explained by the intricate and interconnected signaling pathways that exist between the brain, gut, and adipose tissue and regulate appetite, energy homeostasis, and fat mass maintenance [29–34]. In diabetic rats, the physiological elevation of serum prolactin levels during pregnancy; and postpartum Improves insulin resistance and secretion [35]. Additionally, maternal total energy expenditure increases during milk production by 15–25% [36, 37], resulting in significant postpartum weight loss that improves cardiometabolic status. Despite substantial declines in total body fat mass within 3–6 months postpartum being reported in lactating mothers than in nonlactating [38], studies reported controversial results about lactation affecting body composition and regional fat distribution [38–41].

A direct association between BF duration with ghrelin and protein-peptide YY has been illustrated [42]. By contrast, an adverse association between ghrelin and the risk of MetS and DM was reported [43]. Ghrelin and protein-peptide YY affect metabolism and appetite regulation; ghrelin plays a crucial role in energy homeostasis and glucose regulation, and signals of protein-peptide YY to the brain lead to diminishing food consumption [44–46]. Therefore the possible beneficiary effect of lactation may partly be explained by its effect on ghrelin.

In addition, BF may improve the overall status of mothers through stress reduction. Consequently, it downgrades activation of the hypothalamic–pituitary–adrenal axis and inordinate vagal tones. A significant decline in levels of basal norepinephrine, ACTH, cortisol, and glucose responses to exercise has also been reported in lactating women compared to nonlactating maternal [47].

Previous studies revealed that BF, especially prolonged BF duration, reduces insulin resistance. As a result, each one-year BF duration could be reduced by more than 10% of MetS [48, 49]. It has been shown that suppressing islet menin levels stimulates b-cell proliferation during pregnancy by prolactin to adapt to the dynamic physiological requirements [50]. Notwithstanding their elevated glucose production levels, lower insulin secretion and lower glucose levels were reported in lactating women [51]. Glucose circulating levels have an adverse association with the duration of BF, the effect that remains even many years after stopping BF. It has been shown that serum levels of glucose in women with ≥ 10 years BF are significantly lower than those without lactation [52]; moreover, they reported lower serum triglyceride (0.66 mmol/L vs. 0.91 mmol/L, p = 0.001) and serum cholesterol (4.32 mmol/L vs. 4.78 mmol/L, p = 0.004), as well as a lower waist-to-hip ratio (0.77 vs. 0.81, p = 0.001), in women with prolonged lactation [52]. Although the beneficiary impact of prolonged BF on blood pressure was well documented, The persistence of this effect years after stopping BF is unclear [48, 53, 54].

According to our findings, women with a history of GDM had higher BF duration, exclusive BF, and BF with complementary foods than those without a history of GDM. However, this result contrasts with other studies that reported a shorter period of lactation [55–58]. They assumed that the shorter duration of lactation in women with a history of GDM might be explained through various maternal and fetal morbidities among them [16] that postpone lactogenesis [17] and/or the infants' poor sucking type. It has been shown that the overall rate of exclusively BF duration (37%) in upper-middle-income countries was lower than in others [59]. The longer duration of lactation in GDM women in the present study may be due to the timely prenatal education and intervention that have been available through universal prenatal programs [60]. Besides, supporting BF in the hospital, by health care providers immediately after delivery and during the postpartum period, and by family and community support to persuade BF in Iran is essential [61]. Notably, this purposeful BF support in GDM women is more important and has several advantages for mothers and their offspring.

The thyroid hormone plays an essential role in body metabolism and is one of the several factors influencing body weight [62]. It has been demonstrated that greater levels of thyroid hormones within the normal range are advantageous for metabolism and weight maintenance [63]. A higher thyroid hormone level has been reported in women with a history of GDM associated with a longer BF duration. Findings of research (2018) on women with a history of GDM illustrated that a longer duration of BF can be associated with greater serum fT3 concentrations and fT3:fT4 ratio of 9–16 years postpartum [15] and fT3 is the form of T3 that is in circulation, and the fT3:fT4 ratio is representative of the transformation from T4 to T3. Therefore, the reduction of the incidence of the syndrome in women with a history of GDM may be partly explained through this association; unfortunately, thyroid assessments were not available for the present study.

We found that the beneficiary effect of BF on MetS is enhanced by exclusive BF, so exclusive BF may be more effective in reducing the risk of MetS than total or partial BF among women with a history of GDM. It has been shown that more weight loss occurs following exclusive BF. Furthermore, it is notable that weight loss increases after continuing at least six months of BF [64]. Moreover, women with exclusive BF at 6–9 weeks postpartum had a lower risk for long-term type 2 diabetes mellitus (52% fewer) than those with the exclusive formula [65]. Besides, prior observational studies revealed that an exclusive and longer BF duration might prevent type 2 diabetes mellitus in women with GDM in the future.

Nonetheless, confirmation of these results depends on conducting studies such as longitudinal studies [66]. In this regard, prolactin plays a key role in explaining the beneficial effects of exclusive BF and longer BF duration on MetS incidence in the future [67].

### Limitation and strenght

Our study has several strengths. It was a population-based prospective cohort with approximately two decades of follow-up and several precise measurements of cardio-metabolic risk factors. Also, the accurate measurements and statistical analysis with specific adjustments of influential confounders and conducting an interaction analysis to explore if the effect of BF on MetS is affected by GDM status helped the study reach more powerful results. However, there are several potential limitations that several potential limitations need to be acknowledged. Several confounders, including lifestyle factors and genetic background, have not been addressed in the current study. The history of GDM was self-reported by participants. However, in our country, GDM screening is a routine program conducted as a part of prenatal care. The high sensitivity and specificity of the self-reported assessment of GDM were reported previously. However, another study illustrated the high sensitivity and specificity of GDM through self-report [11]. Our results may be influenced by recall bias secondary to the self-reported duration of BF. Since BF is a crucial period in every woman's life, remembering its duration is reliable even after many years. Although, the results of the research elucidated that maternal recall of BF duration is accurate up to 6 years after birth, and as regards the duration of our follow-ups was three years, it may recall of BF exclusivity is not good at six months after birth [68].

## Conclusion

Our findings showed the protective effect of BF on the incidence of the MetS that increased by prolonged lactation. This effect is more pronounced in those with a longer duration of BF. This beneficial effect was more shown in exclusive BF. Furthermore, a much stronger beneficial effect of exclusive BF duration on the risk of MetS incidence was observed in women with a history of GDM compared to non-GDM. Besides our findings revealed that the promotion and support of continued BF is an opportunity to improve the long-term health of women after a GDM-complicated pregnancy. BF may be considered a simple protective factor that diverts the adverse effects of pregnancy on the lifetime cardio-metabolic status of women. Conducting well-designed comprehensive prospective population-based studies is recommended considering lifestyle and genetic parameters.

## Supplementary Information

**Additional file 1: Table S1.** Unadjusted and multivariable-adjusted* Cox regression analysis for the effect of BF on hazards (95% CIs) of incident MetS in GDM and non-GDM groups. **Table S2.** The Cox regression model explores BF's effect on the hazard of MetS incidence for non-menopausal women. **Table S3.** Cox regression model with interaction effect of BF*GDM on a hazard ratio of MetS for non-menopausal women.

## Data Availability

The data sets produced through the current study are not publicly available but are available from the corresponding author on reasonable request.

## Abbreviations

Mets: Metabolic syndrome
GDM: Gestational diabetes mellitus
BF: Breastfeeding
HR: Hazard ratio
CI: Confidence interval
WHO: World health organization
NCD: Non communicable disease
CVD: Cardiovascular disease
FPG: Fasting plasma glucose
TG: Triglycerides
SBP: Systolic blood pressure
DBP: Diastolic blood pressure
HDL-C: High-density lipoprotein cholesterol
