# Supplementary material for: Lactation duration and lifetime progression to metabolic syndrome in women according to their history of gestational diabetes: a prospective longitudinal community-based cohort study
Source: J Transl Med. 2023 Mar 6;21:177. doi: 10.1186/s12967-023-04005-w (PMC9987076; doi:10.1186/s12967-023-04005-w)
Supplement: Supplementary file 1 — Additional file 1: Table S1. Unadjusted and multivariable-adjusted* Cox regression analysis for the effect of BF on hazards (95% CIs) of incident MetS in GDM and non-GDM groups. Table S2. The Cox regression model explores BF's effect on the hazard of MetS incidence for non-menopausal women. Table S3. Cox regression model with interaction effect of BF*GDM on a hazard ratio of MetS for non-menopausal women. [file 12967_2023_4005_MOESM1_ESM.docx]

**Table S1. Unadjusted and multivariable-adjusted* Cox regression analysis for the effect of BF on hazards (95% CIs) of incident MetS in GDM and non-GDM groups.**

|  | **Non-GDM women** | | | | **GDM women** | | | |
| --- | --- | --- | --- | --- | --- | --- | --- | --- |
| **BF duration** | **Unadjusted model** | | **Adjusted model** | | **Unadjusted model** | | **Adjusted model** | |
|  | **HR (95% CI)** | **p-value** | **HR (95% CI)** | **p-value** | **HR (95% CI)** | **p-value** | **HR (95% CI)** | **p-value** |
| **Total BF** | **0.99(0.99-1.00)** | **0.04** | **0.99(0.98-0.99)** | **0.01** | **0.98(0.97-0.99)** | **0.01** | **0.98(0.97-0.99)** | **0.001** |
| **Partial BF** | **0.99(0.98-0.99)** | **0.04** | **0.99(0.98-0.99)** | **0.003** | **0.98(0.97-0.99)** | **0.02** | **0.98(0.96-0.99)** | **0.003** |
| **Exclusive BF** | **0.98(0.96-1.00)** | **0.1** | **0.98(0.96-1.01)** | **0.2** | **0.93(0.89-0.97)** | **0.003** | **0.92(0.87-0.96)** | **0.001** |
| **^Adjusting variables were age, BMI, family history of diabetes at baseline and physical activity, education, and parity.^**  **^Abbreviation: MetS, metabolic syndrome; BF, breastfeeding; GDM, gestational diabetes mellitus^**  **^Note: Physical activity, education, and parity were included in the model as time-dependent covariates.^**  **^P-value< 0.05 is statistically significant^** | | | | | | | | |

**Table S2.** The Cox regression model explores BF's effect on the hazard of MetS incidence for non-menopausal women.

| variable | Nonmenopausal women | | | |
| --- | --- | --- | --- | --- |
|  | Unadjusted model | | Adjusted model* | |
|  | HR (95% CI) | p-value | HR (95% CI) | p-value |
| Total BF | 0.99(0.98-0.99) | **0.001** | 0.98(0.98-0.99) | **<0.001** |
| Partial BF | 0.98(0.98-0.99) | **0.001** | 0.98(0.98-0.99) | **<0.001** |
| Exclusive BF | 0.96(0.94-0.98) | **0.001** | 0.96(0.94-0.98) | **0.004** |

^* Adjusting variables were age, BMI, family history of diabetes at baseline and physical activity, education, and parity.^

^Abbreviation: MetS, metabolic syndrome; BF, breastfeeding.^

^Note: Physical activity, education, and parity were included in the model as time-dependent covariates.^

^P-value< 0.05 is statistically significant^

**Table S3**. Cox regression model with interaction effect of BF*GDM on a hazard ratio of MetS for non-menopausal women.

| variable | Nonmenopausal women | | | |
| --- | --- | --- | --- | --- |
|  | Unadjusted model | | Adjusted model* | |
|  | HR (95% CI) | p-value | HR (95% CI) | p-value |
| GDM | 2.34(1.54-3.56) | <0.001 | 2.47(1.62-3.75) | <0.001 |
| Total BF | 0.99(0.98-0.99) | 0.01 | 0.99(0.98-0.99) | 0.005 |
| Total BF* GDM | 0.98(0.96-0.99) | **0.02** | 0.98(0.96-0.99) | **0.01** |
| GDM | 2.24(1.48-3.39) | <0.001 | 2.31(1.52-3.49) | <0.001 |
| Partial BF | 0.99(0.98-0.99) | 0.02 | 0.98(0.98-0.99) | 0.004 |
| Partial BF* GDM | 0.98(0.96-0.99) | **0.04** | 0.97(0.96-0.99) | **0.02** |
| GDM | 2.28(1.53-3.40) | <0.001 | 2.52(1.68-3.78) | <0.001 |
| Exclusive BF | 0.97(0.95-0.99) | 0.02 | 0.97(0.95-1.00) | 0.08 |
| Exclusive BF* GDM | 0.93(0.88-0.98) | **0.02** | 0.91(0.85-0.97) | **0.004** |

^*Adjusting variables were age, BMI, family history of DM at baseline and physical activity, education, and parity.^

^Abbreviation: MetS, metabolic syndrome; BF, breastfeeding; GDM, gestational diabetes mellitus.^

^Note: Physical activity, education, and parity were included in the model as time-dependent covariates.^

^The reference group is non-GDM.^

^P-value< 0.05 is statistically significanta^
